# The RALF-FERONIA Signaling Axis: A Central Hub Integrating Plant Growth, Reproduction, and Stress Responses

**DOI:** 10.3390/ijms27010001

**Published:** 2025-12-19

**Authors:** Ekaterina V. Zakharova, Larisa I. Fedoreyeva

**Affiliations:** All-Russia Research Institute of Agricultural Biotechnology, Timiryazevskaya 42, 127550 Moscow, Russia; zakharova_ekater@mail.ru

**Keywords:** RALF, FERONIA, RALF-FERONIA, peptide signaling, plant reproduction, stress response, CrRLK1L

## Abstract

Rapid alkalinization factor (RALF) peptides represent a central class of signaling molecules in plants, regulating processes ranging from fertilization to immune responses. These diverse functions are largely mediated by a conserved receptor complex centered on FERONIA kinase (FER). This review summarizes recent advances positioning the RALF-FER signaling pathway as a major regulatory hub integrating intrinsic and extrinsic signals to coordinate growth, development, and stress adaptation. We examine how this pathway controls the polar growth of root hairs and pollen tubes, orchestrates reproductive barriers and fertilization, and modulates immune and abiotic stress signaling through mechanisms involving ROS, Ca^2+^, and apoplast pH. By framing this new knowledge within the broader framework of known RALF-FER mechanisms, we demonstrate how this pathway achieves high signaling specificity. Finally, we discuss critical unresolved issues and suggest future research directions in the emerging field of molecular stress physiology, highlighting the potential for manipulating this pathway for agricultural crop improvement.

## 1. Introduction

Intercellular communication coordinates physiological processes in plants related to growth, development, and reproduction. Because plants are exposed to a constantly changing environment, intercellular signaling plays a key role in responding to abiotic and biotic environmental influences. Peptides actively participate in the regulation of intercellular signaling processes [1,2,3]. Peptides are small molecules containing 2 to 100 amino acids that, like canonical phytohormones, are capable of transmitting environmental signals over long distances [4,5,6,7]. Due to the great diversity in structure, function and expression patterns, plant peptides are key molecules for rapid initiation, coordination and integration of response reactions [3,5]. Due to the low concentration of peptides in plants and the difficulty of their identification, researchers did not pay attention to them for a long time. However, with the advent of new research methods and the identification of various functions in plant peptides, in recent years there has been a surge of interest in the study of peptides. Based on the study of the functional activity of peptides, they were classified into a special class—the class of peptide hormones [8]. Peptide hormones are usually expressed and secreted in an inactive form; after post-translational modifications, peptides are activated and transported to target cells, where they are able to perform their functions. The identified peptide hormones can be divided into two groups. The first group includes small molecular weight peptides that undergo post-translational modifications, such as sulfation of tyrosine residues, hydroxylation of proline, or arabinosylation [1].

The first functionally active peptide identified was the 18-amino acid SYSTEMIN peptide, isolated from tomato leaves and shown to be involved in the healing of various injuries [9]. Over more than 30 years, various peptide molecules have been identified in plants that play diverse roles in developmental processes, such as CLAVATA3 (CLV3)/EMBRYO ENVIRONMENTAL PEPTIDE (CLE), which is associated with stem cell maintenance; C-TERMINAL ENCODED PEPTIDE (CEP), PHYTOSULFOKIN (PSK), and PLANT PEPTIDE, Sulfated Tyrosine-Containing Peptides (PSY), which are associated with cell proliferation; Root Meristem Growth Factor (RGF), also known as CLE-like or GOLVEN peptide, which is necessary for the maintenance of the root stem cell niche; Caspariam Strip Integrity Factors (CIFs), which are required for Casparium formation [6,10,11,12,13,14].

Despite significant diversity in amino acid sequence, class II cysteine-rich peptides share many common structural features. Peptides in this class have a longer amino acid chain than small peptides, require post-translational modification, are positively charged, and typically contain 4 to 16 cysteine residues, which play an important role in the required conformational folding of the mature peptide. Due to the formation of disulfide bridges, this class of peptides has a relatively fixed structure. Today, the known family of cysteine-rich peptides includes Rapid alkalinization factor (RALF), Epidermal pattern forming factor 1 (EPF1, EPF2), Tapetum determinant 1 (TPD1), Stomatogen/EPF-like 9 (EPFL9), and the signal peptide of the early secretory pathway nodulin [15,16,17,18,19,20].

This review focuses on the RALF family of cysteine-rich peptides, which are important signaling molecules involved in the regulation of various physiological processes, and their interaction with the receptor kinase FERONIA, which is crucial for signaling in response to external and internal stimuli.

## 2. RALF Family

### 2.1. Localization, Functions of RALFs

Rapid alkalization factor (RALF) is a peptide hormone found throughout the plant kingdom that can exhibit both ubiquitous and tissue-specific activity [21]. These peptides were discovered in a peptide hormone assay due to their ability to induce moderate alkalization of *Nicotiana tabacum* cell cultures [15]. However, some members of the RALF family are unable to alkalize the extracellular space of plant cells. RALF peptides belong to a family of cysteine-rich plant peptide hormones that are involved in a variety of physiological and developmental processes, ranging from pollen tube growth to modulation of immune responses [22]. RALF peptides represent an evolutionarily conserved family of peptides, including at least 37 RALF members found in Arabidopsis. RALF peptides with several conserved motifs have been found to be widespread in land plants, highlighting their functional significance [23,24]. RALF peptides are localized in all plant organs. RALF expression is often redundant, and most do not exhibit significant cell or tissue specificity, with the exception of a few that are highly expressed in pollen [21,25].

**Figure 1:**
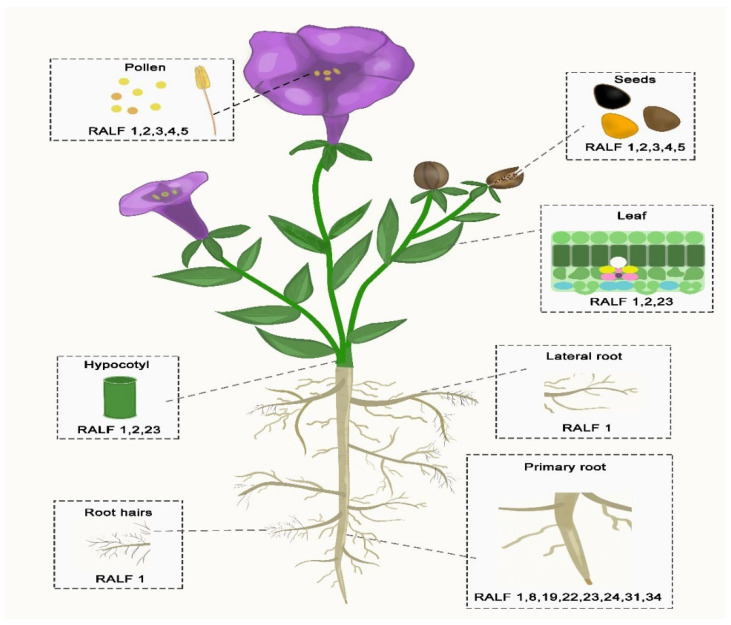
Localization peptides and RALF families in plants.

Based on the data in Figure 1, it follows that some RALF members exhibit tissue-specific localization. Interestingly, A distinctive feature of RALF-1 is that it lacks tissue specificity, yet exhibits the functional activity characteristic of other RALF peptides localized in the same plant organ.

It is interesting to note that homologous sequences of RALF peptides have been found not only in plants. Potential RALF peptides have been isolated from fungal and bacterial genomes [26,27]. F-RALF was isolated from *Fusarium oxysporum*, which, like AtRALF1, caused alkalization and inhibited root growth [26]. Furthermore, it suppressed plant immune responses and enhanced fungal virulence [26]. Plant and non-plant RALF proteins were found to have homologous amino acid sequences in both the C- and N-terminal regions, but the proteins exhibit numerous sequence polymorphisms in the middle domains, which are responsible for RALF receptor affinity [28], which is also pH-dependent and stabilized by disulfide bonds.

RALF was first identified in tobacco leaves as a cysteine-rich polypeptide that also causes an increase in pH [29]. RALF peptides play an important role in various processes, such as root growth and development [30,31,32], root hair formation [33], fertilization [34,35], fruit ripening [36], and plant-pathogen interactions [37,38] (Table 1).

Increased levels of RALF peptides, such as AtRALF1 and AtRALF23, in roots lead to shortening and bushiness of Arabidopsis plants [56,57]. On the other hand, plants with a knockout of this gene, AtRALF1, exhibit root elongation, an increase in the number of lateral roots, and hypocotyl elongation [31,32,58]. The Arabidopsis RALF34 peptide is thought to act as a negative regulator of lateral root initiation, likely localizing near existing primordia [31] and acting extracellularly and autonomously, similar to CLE peptides [8,59]. RALF peptides and their receptors form a signaling system involving many different signaling pathways and molecules [16,60,61]. However, the role of each participant in the RALF signaling pathway is not yet fully understood.

### 2.2. RALF Peptide Homology

**Figure 2:**
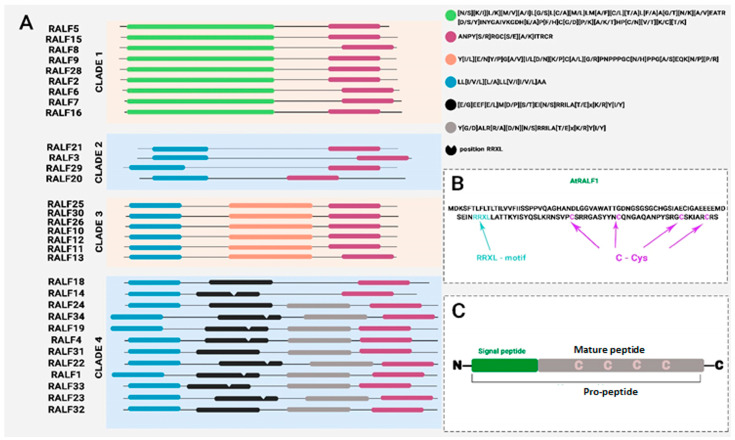
Primary structure of RALF peptides. (**A**)—Homologous motifs in the RALF peptide family. (**B**)—Primary sequence of the RALF1 peptide. (**C**)—Structure of the RALF1 peptide precursor.

RALF peptides exhibit high primary structure homology (Figure 2A). The peptides share 2 to 4 homologous motifs, allowing them to be grouped into 4 clades. The primary structure homology found across diverse plant species suggests that RALF peptides play a fundamental role in many plant families.

The N-terminal YISY motif of the mature peptide is required for efficient binding of RALF peptides to their target receptor [62]. The terminal Tyr and internal I/L of this motif are the most highly conserved. However, some RALF family members exhibit a substitution of Ser for Gly or Thr. This substitution is likely related to their receptor affinity and functional activity. Homologous GASYY motifs and C-terminal RCRR(S) motifs have been found in many mature AtRALF sequences, but their roles are currently undefined. Most AtRALF peptides contain four cysteine residues, which are positionally conserved, determine their spatial structure, and are therefore required for functional activity (Figure 2B) [6,16]. Cysteine oxidation has been shown to be accompanied by altered peptide folding, which disrupts physiological activity. The formation of disulfide bridges in peptides results in the formation of peptide loops, but due to the close spacing of the cysteine residues, these loops are short, favoring the disordered conformation of the peptides in the free state [16]. Structural analysis of AtRALF8 indicates that the peptide is almost completely unstructured [63]. This disordered conformation of RALF peptides is likely important for binding to various receptors and ligands, which cause the RALF peptide to adopt a strictly defined structure upon binding.

Based on amino acid sequence analysis, four peptide clades with the highest homology can be identified (Figure 2). Interestingly, peptides from clade 4, which includes RALF1, exhibit high functional activity compared to other clades. Although RALF peptides share some structural features, there is no clear relationship between tissue localization and functional activity of the peptides and their amino acid structure (Table 1). This demonstrates that even small amino acid substitutions in the peptide structure are important for functional activity. For example, substitution of Ile-6 with Ala leads to a loss of alkalizing ability and inhibition of root growth in tomato RALF [62]. It was also noted that a seven-amino acid difference between AtRALF4 and 19 is important for alkalizing activity [64].

RALF peptides are processed from larger precursors containing up to 160 amino acids. The precursor of the RALF peptide is a preproprotein containing a long secreted peptide (Figure 2C). Since RALF peptides contain a secreted peptide, this indicates that the peptides are secreted and localized in the apoplast [16,65]. Using a chimeric fluorescent protein RALF–GFP (green fluorescent protein), it was shown that RALF peptides are localized not only in the apoplast but also in the endoplasmic reticulum [65].

The RALF peptide precursor is digested by proteases of the subtilase family, resulting in the formation of a mature active peptide located at the C-terminus of the precursor [66,67]. An Arg-Arg (RR) motif has been identified in the primary structure of RALF family peptides, which may serve as a potential cleavage site for proteolytic enzymes [16]. A unique proteolytic enzyme (AtS1P, SITE-1 PROTEASE) has been identified that cleaves the proprotein at the RR site, separating the RALF peptide from the signal peptide, forming the active peptide [24,67]. AtS1P protease has been shown to be localized to the Golgi apparatus. Based on this finding, it has been proposed that RALF peptide precursors are initially processed in the Golgi apparatus and then translocated to the apoplast via the secretory pathway [68]. However, the protease can migrate to the apoplast, where the signal peptide is cleaved, forming the active mature RALF peptide.

RALF peptides are widespread in the plant kingdom and have been found in monocots, dicots, and gymnosperms [16]. Among higher plants, the number of RALF peptides in *Arabidopsis* reaches at least 37 members. This is believed to be due to tandem duplication. It is estimated that 50% of the peptides in *Arabidopsis* arose through tandem duplication, while in rice, the figure is 43.7% [69]. Furthermore, the number of RALF genes in poplar, for example, is 30% lower than in *Arabidopsis*, despite the genome being 67% larger [23]. Phylogenetic analysis of RALF peptides in *Arabidopsis*, poplar, maize, and rice identified six distinct conserved amino acid motifs within the family (Figure 2). Based on these data, ten distinct gene groups were created. It is possible that some genes have evolved under positive selection, causing changes in protein sequence, resulting in some RALF peptides lacking certain conserved motifs [24].

The widespread localization of RALf1 in all tissue types and its broad spectrum of functional activity, unlike other RALF family peptides, suggests that other RALF family members complement the activity of RALf1 when its activity is suppressed.

## 3. Receptor Kinase FERONIA

The diverse and numerous functions of RALF peptides depend on interactions with intercellular receptors. Membrane receptor-like kinases regulate kinase activity to initiate signaling through differential phosphorylation/dephosphorylation at specific protein sites [59]. The *Catharanthus roseus* family of receptor-like kinase 1-like proteins (CrRLK1L) play a key role in maintaining cell wall integrity, intercellular communication, and responses to external stressors [70,71]. CrRLK1L, like all membrane receptor-like kinases, consists of a transmembrane domain, an intracellular kinase domain, and an extracellular malektin-like domain [72,73]. CrRLK1L proteins are a subfamily of RLKs and structurally contain two malectin-like domains (MA and MB) in the extracellular domain, a transmembrane domain (TMD), and an intracellular kinase domain (KD). Malectin is a diglucose-binding protein located in the endoplasmic reticulum in mammals [74].

**Figure 3:**
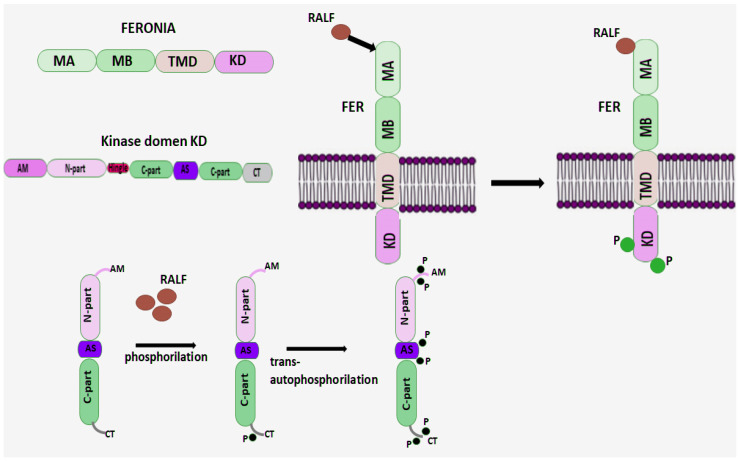
Schematic diagram of FERONIA (FER) fragments: MA and MB—malektin-like domains A and B; TMD—transmembrane domain; KD—intracellular kinase domain; diagram of KD fragments: AM—juxtamembrane domain; N-part; C-part; AS—activation segment; CT—C-terminal region. (**Upper right panel**): location of FER kinase complex fragments in the cytoplasmic membrane. Activation of FER phosphorylation upon binding to the RALF peptide. P–phosphate groups. (**Lower panel**): the process of activation of phosphorylation and trans-autophosphorylation.

FERONIA (FER) is the best-known member of the CrRLK1L family, to which the secreted peptide RALF, which is localized extracellularly, can bind via its extracellular domain [6,75] (Figure 3). Once the FER-RALF complex is formed, a cascade of signaling reactions is triggered that regulate numerous cellular processes [7,76].

FER is a Ser/Thr protein kinase. All Ser/Thr and Tyr protein kinases have a catalytic domain of approximately 300 residues in which the active site is located between an N-terminal domain consisting of a β-sheet and one α-helix and a larger C-terminal domain, which are connected by a linker [51]. The C-terminal portion consists predominantly of α-helices and a 20–35-residue activation site located between a conserved DFG motif and an APE motif. FER contains four conserved domains, like all Ser/Thr protein kinases. The presence of a malectin-like domain, which has homology to the carbohydrate-binding malectin protein of *Xenopus laevis*, suggests that FER ligands can also be glycosylated, which significantly expands the list of possible signaling molecules [77,78]. Furthermore, the FER family has acquired the ability to bind polysaccharides via the malectin domain, allowing these receptors to function as cell wall sensors. Interaction with the cell wall significantly expands the capabilities of FER as a receptor, enabling it not only to detect cell wall disturbances but also to receive and transmit signals from cytoplasmic processes [70,78,79].

It is proposed that FER, as a Ser/Thr receptor kinase, is activated by autophosphorylation [80] and also exhibits kinase activity in vitro both toward itself and toward synthetic substrates (Figure 3) [81,82]. Studies on the requirement for FER receptor phosphorylation reveal the importance of this modification for function, although in some cases phosphorylation is not necessary. It is proposed that the FER receptor recognizes a ligand and phosphorylates another protein, triggering a signal transduction cascade [83]. When Lys is replaced by Arg in the active site of FER kinase, a loss of kinase activity is observed, forming the so-called “dead” FER kinase, which is, however, able to compensate for fer-1 (null phenotype) and shows that in some cases phosphorylation is not necessary for FER function during transmission to the pollen tube (PT) [84,85]. On the other hand, “dead” FER kinase resulted in a decrease in the root response in *fer* mutants and calcium mobilization induced by RALF1 in the cytoplasm compared to the wild-type FER in *Arabidopsis* [84]. These data confirm that FER kinase activity is necessary for the manifestation of the root growth inhibition function involving the RALF1 peptide. However, it was suggested that other kinases that are able to compensate for the kinase activity in *fer* mutants may also be involved in the phosphorylation process. Therefore, further study is required to confirm the requirement for kinase activity in FER.

FER functions as a dual-specificity kinase: it not only phosphorylates the amino acid residues in the active site required for its activation but can also phosphorylate multiple downstream substrates. This demonstrates the important role of phosphorylation in these processes. However, the specificity of FER interactions with various substrates and how these interactions control specific signaling without affecting other pathways remain unclear. One possible explanation is that this may be due to the diversity of phosphorylation sites, the degree of FER phosphorylation, or the cellular specificity of substrate factors.

Chakravorty et al. [86] obtained interesting data that may offer promising avenues for further research. They found that different levels of expression of the inactive FER kinase resulted in the formation of distinct rosette phenotypes and differences in RALF-induced stomatal movements. However, the mechanism by which these phenotypes are affected by dose requires further study.

In addition to phosphorylation, FER is also regulated transcriptionally or post-translationally by many intrinsic and extrinsic factors. At the transcriptional level, FER is induced by treatment with BR and ethylene [87,88]. Moreover, *FER* expression is also regulated; for example, BZR1 induces *SlFER2/3* expression by binding to the promoters of genes that mediate RBOH1-dependent ROS generation and BR-regulated heat tolerance in tomato [89,90]. Furthermore, the MADS-box transcription factor RIN binds to the *SlFERL* promoter, activating its expression, which mediates ethylene-regulated fruit ripening [90]. At the post-translational level, FER activation and phosphorylation are promoted by RALF and ABA [39].

Another important mechanism regulating FER kinase activity is its dephosphorylation. It has been determined that the phosphatase ABI2 can directly interact with FER and dephosphorylate it. As a result of dephosphorylation, inhibition of FER activity is observed [39]. This dependence on phosphorylation underlies the mechanism by which plants regulate their response to abiotic stresses through the interaction of ABA and RALF1 [39]. In particular, in the ABA signaling pathway, its negative regulator ABI2 interacts with FER and dephosphorylates it, suppressing its activity. It has also been found that the receptor kinase ERU is able to decrease the phosphorylation level of FER and increase the number of phosphorylated residues in H^+^-ATPase 1/2, which is involved in auxin-regulated root hair growth [7,91,92]. Thus, given the key functions of FER in plant growth and development, it is worthwhile to study the factors involved in FER transcription and post-translational modifications, which will provide new insights into the cellular processes regulated by FER.

## 4. FERONIA-RALF Interaction

FER proteins exhibit functional diversity, complexity, and cell type-specificity. FER kinase can exhibit all of these properties depending on specific downstream signals. Guanine nucleotide exchange factor GTPases, including GEF1, GEF4, GEF7, and GEF10, have been identified as downstream factors. FER acts as an upstream regulator of RAC/ROPs by directly interacting with and activating GTPases. Activated RAC/ROPs interact with several different families of signaling mediators, including NADPH oxidases and ABI2 phosphatase, to regulate various cellular processes, including plasma membrane rupture, root hair development, and the regulation of ABA and auxin signaling [93,94,95]. FER is also involved in the regulation of JA signaling by phosphorylating and destabilizing myelocytomatosis 2 (MYC2) protein, thereby negatively affecting the immune response [52].

Haruta et al. [6] reported that RALF1 specifically interacts with the extracellular domain of FER, leading to FER activation. FER then phosphorylates H^+^-adenosine triphosphatase 2 (AHA2) in the plasma membrane at Ser899, suppressing primary root cell elongation in *Arabidopsis*. Based on these data, it can be suggested that RALF1 activates FER by triggering FER phosphorylation at specific sites (such as S871, S874, or S858). Later, Du et al. [96] revealed the interaction of RIPK with FER, which triggers the phosphorylation of both FER and RIPK, resulting in the suppression of root growth in response to RALF1. It was suggested that FER activation and phosphorylation may be mediated by FER–RIPK interaction and, consequently, interdependent phosphorylation [96]. Thus, the diverse functions of RALF in FER signaling and kinase activation may be explained by FER interacting with other factors outside the cell, such as LRE-like GPI-AP1 (LLG1) and leucine-rich repeat extensins (LRX), as well as with various factors inside the cell, such as RIPK and GEF.

To perform its biological functions, RALF binds to its receptors on the cell membrane [6,43,46,50,96]. Several molecular mechanisms underlying RALF recognition by CrRLK1L receptors and subsequent receptor activation have been proposed [28,48,97]. Some molecular mechanisms underlying the recognition of RALF by CrRLK1L receptors and subsequent receptor activation have been proposed [28,48,97]. However, only in the case of RALF8 has the interaction of the peptide with its main receptor FER been studied in detail. RALF8 has been shown to be disordered in solution, with the exception of an ordered ring located at the C-terminus, formed due to disulfide bonds. It has been suggested that only upon binding to the receptor does RALF adopt an ordered structure [63]. Studies Du et al. [98] and Moussu et al. [28] revealed the existence of a deep cleft in the CrRLK1L receptor between two extracellular malectin-like domains formed by highly conserved aromatic and polar amino acid residues, which may be a potential binding site for RALF with the receptor.

Crystal structure analysis of the RALF-receptor complex reveals that various motifs within RALF can be involved in binding to their respective receptors. For example, the N-terminus of RALF23 forms an α-helix that fits into the groove on the surface of the LLG2–FER complex, while the YISY motif at the N-terminus of RALF23 interacts with LLG2 via a hydrophobic polar structure. When binding to FER via its C-terminus, RALF23 is able to increase the stability of the complex [97]. Covalent cross-linking and mass spectrometry have shown that the extracellular domain of FER binds to the conserved C-terminus of RALF1, and deletion of the YISY motif in RALF1 results in a 50% decrease in binding affinity to FER [99]. This finding indicates that the YISY motif at the N-terminus of RALF is involved in the formation of α-helical structures that are critical for LLG and FER binding [97,99].

As demonstrated for RALF4, for strong binding of the peptide to the receptor, the disulfide bond must be located on the inner surface [28,43]. After analyzing the structure of the ligand-receptor complexes formed by RALF and its binding receptors, the researchers concluded that RALF forms complexes with ligand-induced receptors through various mechanisms [28,97]. One of the mechanisms is the participation of co-receptors in the formation of the ligand-receptor complex. Various proteins involved in RALF recognition and binding have been identified as CrRLK1L co-receptors [43,48,97], such as the LRE protein and its homolog LLG, which promote the structural stabilization and modification of FER, thereby controlling the location and timing of FER signaling [97,99,100,101]. The LLG1 protein acts as a co-receptor for FER and helps transport FER from the endoplasmic reticulum to the cell membrane surface [97,100]. Xiao et al. [97] confirmed that RALF23 promotes the formation of the LLG–FER receptor complex using analytical ultracentrifugation and co-immunoprecipitation assays. Another immune response receptor (LRK) binds to RALF and enhances the recruitment of immune recognition complexes to BAK1 [48]. These data may indicate the involvement of different co-receptors in the assembly of RALF with the FER receptor for subsequent signaling.

## 5. RALF-FER Signaling

To thrive in a constantly changing environment, plants must recognize, coordinate, integrate, and transmit multiple internal and external signals to coordinate growth, development, and stress responses. Receptors that respond to environmental changes play a key role in coordinating this process. In recent years, the FERONIA receptor kinase in the model plant *Arabidopsis* has emerged as a leading candidate for regulating numerous cellular and molecular responses that ensure growth and survival [102].

### 5.1. ROS in FER-RALF Signaling

Abiotic stressors generate ROS, causing oxidative stress in most plants [103]. Redox homeostasis in plants is a normal state in which an efficient defense system maintains the correct balance between ROS production and removal [104]. The basal amount of ROS is crucial for the signaling process [104,105]. Low levels of ROS trigger signaling processes that alter normal plant metabolism, while excess ROS causes oxidative damage to cells [105,106]. They are mainly produced in the apoplast by NADPH oxidases (called respiratory burst oxidase homologs; RBOH) and some oxidases and peroxidases, as well as in the chloroplast, mitochondria, peroxisomes, and possibly other cellular compartments through various pathways [107,108,109,110,111,112]. Various abiotic stresses and/or various combinations of abiotic stresses (stress combinations) likely induce the formation of distinct ROS signatures in plant cells, and the decoding of these signatures by various ROS sensors can generate a stress-specific signal.

ROS signals are decoded through various redox reactions involving sulfur-containing protein residues (e.g., the -SH group of cysteine). The formation of disulphide bonds in proteins alters protein structure and function, which allows for the regulation of transcription factor binding to DNA and influences transcription [113,114].

**Figure 4:**
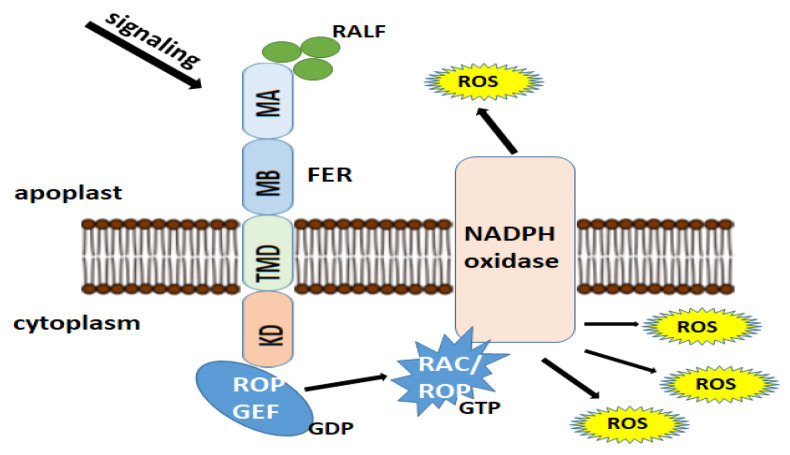
Schematic representation of the role of RALF-FER in the ROS signaling pathway. RALF increases FER phosphorylation, which leads to activation of the ROS production pathway via the GEF1/4/10–ROP11/ARAC10 pathway.

The redox state of cysteine residues in proteins and peptides can play an important role in plant stress responses (Figure 4) [115]. CyS metabolic pathways in plant cells remain poorly understood. Olm et al. [116] suggested that molecules such as glutathione and enzymes such as glutathione reductase (GR), thioredoxin reductase, and glutaredoxins (GRX) may be responsible for the reduction of CySS. In protozoa, thioredoxin reductase performs this function [117], but several studies of glutathione reductase (GR) have shown that this enzyme is unable to reduce CySS [118,119].

Stegman et al. [48] demonstrated the involvement of *Arabidopsis* RALF peptides in the regulation of intracellular ROS levels. They observed a decrease in the relative levels of NADH dehydrogenase, complex I of the respiratory chain, and succinyl-CoA synthase, the enzyme that converts succinyl-CoA to succinate. Thus, these results demonstrated an important role of RALF in the functioning of the respiratory chain. Moreover, it also indicated a negative role of RALF in the activity of complex III. A decrease in the activity of the respiratory chain is accompanied by a decrease in the electron flux, which leads to a decrease in the rate of superoxide ion formation [120,121]. It was also noted that roots with increased RALF expression had a decrease in the H_2_O_2_ content compared to control samples. Since superoxide anions are converted into H_2_O_2_ by superoxide dismutase, the decrease in H_2_O_2_ levels in roots can be explained by the inhibition of electron transfer in the respiratory chain. It is suggested that RALF can directly influence ROS production [122].

One mechanism explaining the role of FER in ROS accumulation is its regulation by RHO-GTPase (RAC/ROP) and NADPH oxidase (Figure 4). ROS levels are increased through the interaction of FER with RHO-GTPase—guanine nucleotide exchange factor (ROPGEF) [93,123,124,125]. RAC/ROP is involved in the activation of NADPH oxidase, which, in turn, initiates ROS generation and ROS-dependent processes [93,100,126].

This finding is consistent with the work of Song et al. [53], who showed that a mutation in the FERONIA gene (FER), the RALF receptor in *Arabidopsis*, was associated with lower levels of ROS production. Thus, it is likely that RALF may directly influence ROS production. Moreover, since Calcium-dependent protein kinases (CDPK) is activated by interaction with Ca^2+^ ions [122], increased expression of this kinase may result in increased root sensitivity to intracellular calcium levels. When treated with nanomolar concentrations of natural or synthetic RALF peptides, cytoplasmic Ca^2+^ levels peaked within 40 s. This suggests that different RALF peptides may act in concert: some RALF peptides may bind to the RALF receptor and initiate Ca^2+^ release, while others may increase sensitivity to ABA. Finally, it is important to note that Ca^2+^ levels can fluctuate widely even over short periods of time. The interaction of these dynamically changing Ca^2+^ levels with RALF-mediated responses may underlie RALF’s multiple effects.

Searches for molecular interactions with ROPGEF1 revealed that FER is a cell surface receptor for RAC/ROP, which is a global signaling switch. RAC/ROP acts on multiple effectors, influencing various response systems in the cytoplasm, including Ca^2+^ dynamics, ROS, actin, microtubules, and vesicular transport. All of these are fundamental components of cellular activity [93,127,128]. For plant growth and reproduction, FER-controlled ROS production is critical for the growth of polarized root hairs, the release of sperm from pollen tubes in the female gametophyte, and the function of the stigma “gating” that senses and discriminates between desirable and undesirable mates [93]. During pollen tube elongation, strict regulation of ROS, which is mediated by ANXUR and BUPS, is essential for pollen tube integrity during growth [98,129,130].

The FER-RAC/ROP-ROS pathway plays a key role in proper plant development. Arabidopsis has more than 10 members of the ROPGEF and RAC/ROP protein families, as well as a family of activated GTPase effectors, including ROS-producing NADPH oxidases, which are versatile signaling mediators in numerous physiological processes [131,132,133]. Thus, the regulation of the RAC/ROP GTPase switch and ROS are universal hubs for signaling diversification (Figure 4), contributing to the wide range of biological functions of FER.

Using CsRALF34, as an example, it was shown that the peptide negatively affects the activity of complex III, leading to electron deficiency in the respiratory chain and, accordingly, to a decrease in the rate of superoxide formation, as well as a decrease in H_2_O_2_ levels. Based on the data obtained, it can be assumed that the CsRALF34 peptide promotes the activation of the antioxidant defense system [134]. Thus, it can be concluded that CsRALF34 is critically important for the suppression of H_2_O_2_ production and the activation of multiple cellular antioxidant systems. It can be hypothesized that CsRALF34 mediates these effects through signaling cascades.

### 5.2. RAL-FER-PECTIN

**Figure 5:**
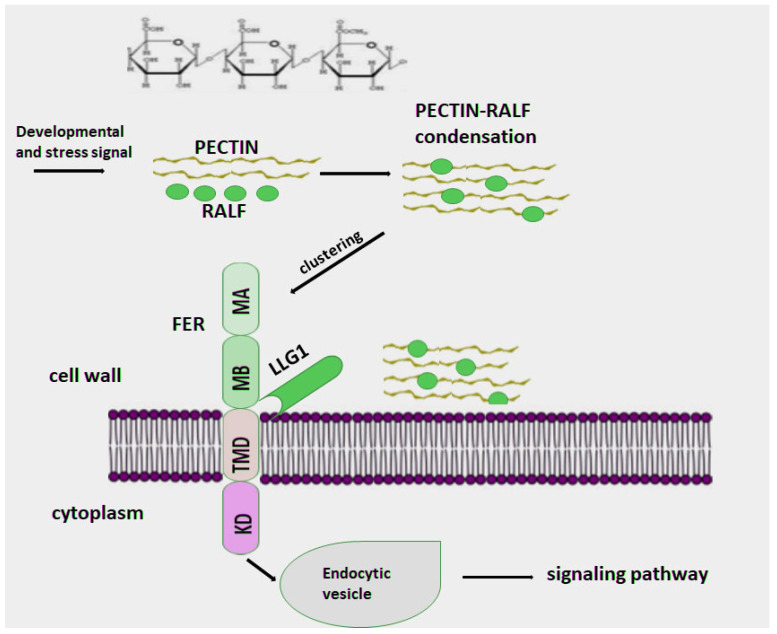
Separation of pectin into liquid and solid phases via RALF-FERONIA signaling. Stress and developmental signals lead to pectin demethylation and the formation of a pectin-RALF1 condensate. The resulting RALF1-pectin condensates localize to the plasma membrane as microdomains, activating the FER and LLG1 receptors, which induce endocytosis. The formation of endocytic vesicles activates multiple signals, such as Ca^2+^, ROS, and ROP, which are involved in regulating plants’ development and their stress response.

The plant cell wall is the first barrier to external factors. It is closely associated with the cell membrane and influences its biological activity [135,136]. Pectin is a cell wall polysaccharide that plays an important role in maintaining the structure and integrity of the cell wall and provides its plasticity to support various biological processes [137,138,139,140]. Pectin is a linear polymer of α-1,4-linked galacturonic acids (homogalacturonan) (Figure 5). It is synthesized in the Golgi apparatus as methylgalacturonan [141]. In the cell wall, pectin is present both in its native methylesterified form and with varying degrees of deesterification. In the apoplast, cross-linking of the free carboxyl groups of deesterified pectin occurs when it binds to Ca^2+^ ions, thereby providing rigidity and permeability to the cell wall [136,137,138,139,142]. The extracellular domain of FER is capable of binding to pectin [111,112], affecting the state of the cell wall and thereby controlling many biological processes [143,144,145]. FER receptor kinase and RALF also interact with extensins. Extensins are hydroxyproline-rich glycoproteins that play an important role in the structural integrity of the cell wall by forming inter- and intramolecular cross-links with the cell wall matrix [146]. It has been shown that mutants with loss of FER exhibit a weakened cell wall. For example, roots in such phenotypes grow slower because they are less able to overcome barriers than their wild-type counterparts [147]. It was also noted that high salt content led to the cessation of root growth (Figure 5) [144]. The FER cell surface signaling module consists of three main components. FER interacts with a glycosylphosphatidylinositol-anchored protein (GPI-AP) of the LORELEI-like GPI-AP (LLG) family [101]; together, they function as a co-receptor pair for signal perception [94,100,127]. RALF peptides bind to the FER-LLG1 receptor pair and regulate signaling outside the cell [61,148]. To transmit signals to the cytoplasm, FER directly interacts with ROPGEFs—guanine nucleotide exchange factors that activate RAC/ROPs, RHO GTPases that regulate a variety of processes [128,131,132,133].

In response to environmental changes, pectin is capable of undergoing phase transitions [106,149,150]. Phase separation of pectin, which occurs on the cell surface, plays an important role in the recognition and binding of extracellular signals, providing a wide range of functions for FER-LLG1. Researchers [151] have shown that upon binding of RALF and FER-, receptor clusters are formed on the cell surface, which is the initial step in activating signal transduction. Upon receptor clustering, membrane curvature toward the inside of the cell is observed, which promotes stimulation of ligand-induced endocytosis [152,153,154,155,156]. A dependence of RALF signaling in the FER-LLG1 signaling pathway on the degree of deesterification of pectin was discovered. Pectin deesterification, as well as its modification and destruction, is carried out by many agents [138,149,157,158]. These reactions involve, for example, pectin methylesterases (PMEs) located in the cell wall, which cleave the diester bond, and polygalacturonase ADPG1 [138,159] cleaves deesterified pectin into pectin fragments of different sizes [160].

Stress is a negative factor that inhibits plant growth [161]. It is known that endocytosis causes a change in the density of the cell membrane, which promotes the processing of the damaged surface of the cell membrane, as well as the transmission of signals caused by these damages [152,162,163,164,165]. Thus, massive endocytosis mediated by pectin-RALF-FER-LLG1 can function as a mechanism of adaptation to stress [164,165,166]. Using mutant plants, it was shown that FER protects against the effects of high salt concentrations [144,157], moreover, wild-type phenotip is able to resume growth after adaptation to high salinity conditions [157]. The reduced tolerance of *fer-4*, *llg1-2*, *PMEI5*, and *ralf1-3* mutant seedlings to stress factors was reflected in their increased sensitivity to growth under prolonged exposure to high salt concentrations, which proves that and elevated temperature. These results together suggest that pectin-RALF-FER-LLG1 plays a concerted and critical role in plant stress tolerance through endocytosis.

The synthetic peptide RALF1 has been shown to bind to cell wall pectin and trigger endocytosis, inhibition of root growth, Ca^2+^, and ROS burst [167,168]. It has been shown that the peptide does not penetrate into the cell during endocytosis, but remains outside the cell due to its binding to extensin molecules. Thus, it is likely that extensins promote the formation of pectin-RALF1 condensates. This fact allows RALF1 to cluster FER and LLG1 receptors in membrane subdomains and activate the FER-LLG1 complex by binding to the receptors via its homologous YISY domain [28,167,168].

Environmental factors such as high temperature and salinity were found to increase the levels of RALF and short fragments of demethylated pectin. As a result of this exposure, pectin-RALF1-FER-LLG1 and pectin-RALF-LRX condensates were formed, respectively. The formed condensates activate the RALF signaling pathway, which ultimately triggers rapid adaptation processes and physiological processes for optimal plant development [168].

## 6. RALFs Control Hybridization Barriers in Plants

**Figure 6:**
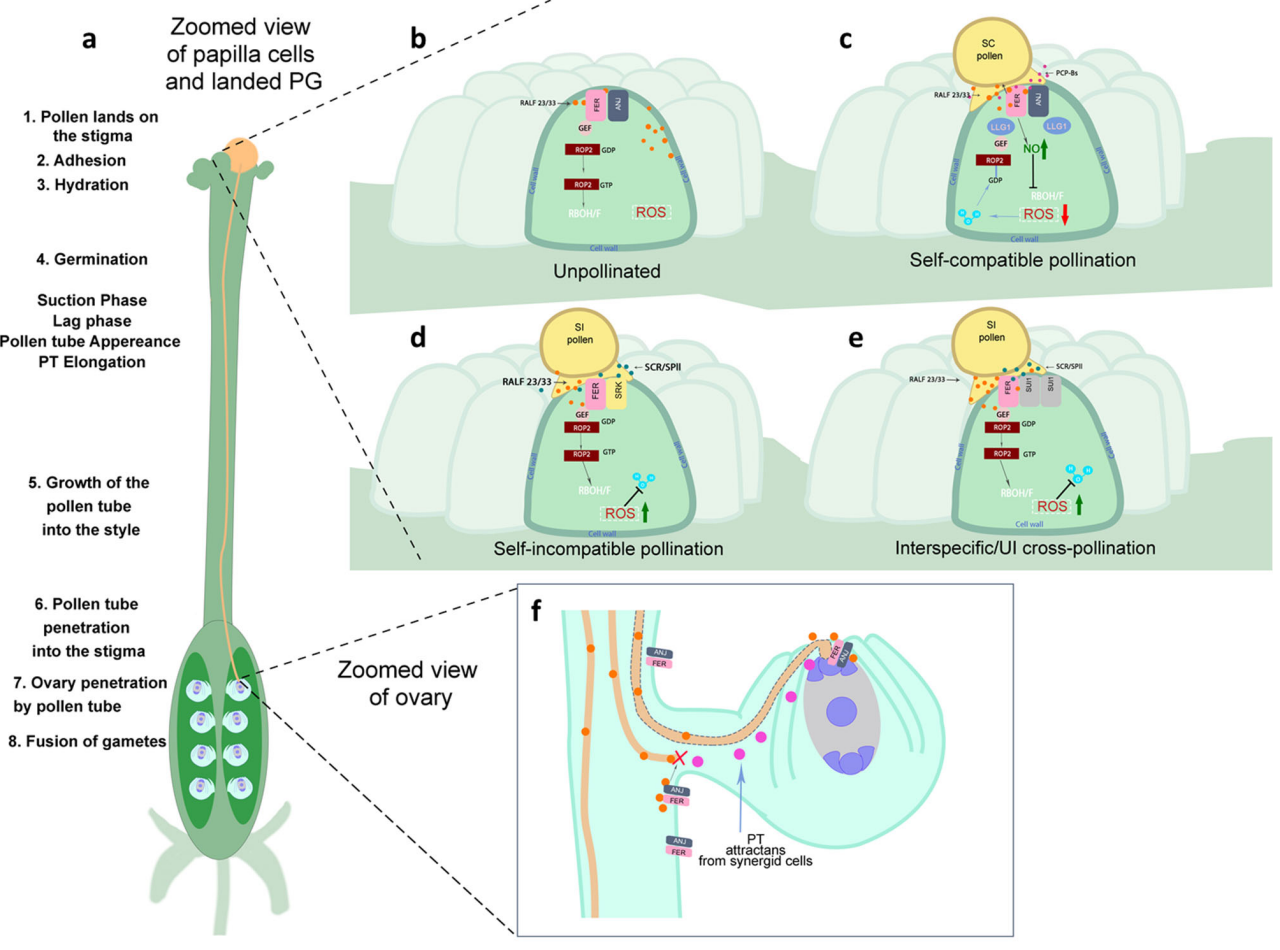
A model of pollen grain hydration during the functioning of hybridization barriers in *Brassicaceae*. (**a**)—Stages of male and female gametophyte interaction in the pollen-pistil system; (**b**)—Unpollinated pistil. In the absence of pollen on the surface of the papilla, the transport of water and reproductive materials is suppressed. This is achieved through the continuous secretion of autocrine signaling ligands RALF23 and RALF33, which are perceived by the stigmatic ANJEA/FERONIA signaling complex; (**c**)—Self-compatible pollination. In the case of compatible pollination, the PCP-B protein from the pollen coat competitively binds to the LLG1/ANJEA/FERONIA signaling complex on the papilla surface. This competitive inhibition switches off RBOHD-dependent ROS production, resulting in decreased intracellular ROS levels, which allows the transport of water and reproductive materials to facilitate pollen hydration; (**d**)—Self-incompatible pollination. Upon attachment of incompatible pollen, the transport of water and reproductive materials within the stigmatic papilla is suppressed. This is achieved through the continuous secretion of autocrine signaling ligands RALF23 and RALF33, which are perceived by the stigmatic LLG1/ANJEA/FERONIA signaling complex. This interaction activates the membrane-localized NADPH oxidase RBOHD via the Rop2 GTPase pathway, subsequently promoting ROS production in the cell wall, which are transported back into the cytoplasm of the stigmatic papilla, blocking the transport of water and reproductive materials; (**e**)—Model of preventing pollen hydration during interspecific/unilaterally incompatible (UI) cross-pollination. In interspecific or unilaterally incompatible pollen, SRK activates FER-mediated ROS production and an ARC1-dependent degradation pathway, inhibiting pollen hydration; (**f**)—Model of the effect of pollen-specific RALFs on female tissues located in the FER, ANJ, and HERK1 receptor complexes for the coordinated establishment, maintenance, and removal of the polytubey block during fertilization.

In recent years, significant progress has been made in elucidating the mechanisms of pollen-pistil interactions, providing a deep understanding of these selective phenomena. Recent advances in revealing the role of RALF family peptides represent an important step forward in identifying specific molecular players, but they raise new questions regarding the reasons for the high apparent redundancy of these proteins. It is now undeniable that the RALF peptide family is crucial for regulating male gametophyte growth and fertilization (Figure 6).

### 6.1. Intraspecific Reproductive Barriers Self-Incompatibility

Self-incompatibility (SI) is a prezygotic reproductive barrier used by hermaphroditic plants to prevent self-fertilization and promote outcrossing [169]. During pollination/fertilization, the male gametophyte undergoes several stages after landing on the pistil stigma (Figure 6a): adhesion, hydration, pollen tube (PT) germination, PT growth through the pistil’s transmitting tissues, penetration into the ovary, and fertilization. Reproductive barriers can function at all these stages.

Members of the *Brassicaceae* family, particularly *Arabidopsis*, are characterized by a sporophytic type of SI and have dry stigmas [170]. In such plants, the critical point for the functioning of the SI mechanism and interspecific/intergeneric hybridization barriers (IRB) is hydration after pollen grains (PGs) land on the stigmatic papillae [171]. Following this, the papilla cell, located in the area constricting the PG attachment site, releases compatible factors via polarized secretion. As a result, the secreted fluid is absorbed by the dry PGs through an efficient channel created by the transformation of the pollen coat lipids, thereby promoting the metabolic reactivation of PGs (Figure 6) [167].

A “lock and key” mechanism involving the binding of stigmatic RALF (sRALF) and pollen RALF (pRALF) with FER, a *Catharanthus roseus* receptor-like kinase 1-like (CrRLK1L) receptor, was recently discovered in *Brassicaceae* during the functioning of the hybridization barrier [171,172]. In the absence of pollen attachment, the transport of water and reproductive materials within the stigmatic papilla is suppressed. This is achieved through the continuous secretion of autocrine signaling ligands RALF23 and RALF33, which are perceived by the stigmatic ANJEA/FERONIA signaling complex (Figure 6b).

Compatible pRALFs act as a key, allowing the pollen tube to germinate, leading to successful fertilization (Figure 6c). In contrast, in the absence of compatible pRALFs, sRALFs bind to FER, inducing a “lock” state that prevents pollen tube penetration (Figure 6d). Despite the crucial role in Brassicaceae hybridization, the structural basis of sRALF binding to FER remained unclear for a long time due to the high flexibility of RALF peptides. Bhalla et al. [172] showed that pRALF and sRALF bind to negatively charged regions in FERONIA with different binding affinities. The binding of RALF (stigmatic and pollen) to FERONIA is highly dynamic and dose-dependent, requiring higher doses of pRALF to overcome the hybridization barrier created by sRALF and FER [171].

To date, a major role in the process of PT growth and arrest is attributed to ROS. In *Arabidopsis*, accumulation of ROS in the papillary cell prevents pollen hydration. ROS production is induced by RALF23/33 peptides produced by the papilla, which are perceived by two stigmatic receptor-like kinases, ANJEA (ANJ) and FER of the Catharanthus roseus RLK1L-like kinase family (CrRLK1L). POLLEN COAT PROTEIN B-class (PCP-B) from the pollen coat causes a reduction in ROS levels in the wild-type stigma papilla after compatible pollination. PCP-B and RALF compete for binding to the ANJ-FER complex in a dose-dependent manner, leading to reduced ROS production, thereby promoting hydration of compatible pollen and establishing a locking mechanism to regulate ROS production via a downstream executor, the classic Rho-like GTPase-regulated NADPH oxidase RBOHD pathway (Figure 6c) [54,140]. Furthermore, PCP-B was shown not only to competitively bind FER, interrupting RALF33-mediated ROS production, but also to trigger rapid NO production to nitrosylate FER, inactivating Rac/Rop-regulated RBOH-dependent ROS production (Figure 6c) [173].

However, the cellular-level signaling mechanisms that follow the binding of sRALF and pRALF to the FER receptor-like kinase associated with the pollen membrane on the stigma remain unclear. Somoza et al. [174] showed that adding synthetic RALF4 peptide rapidly stops pollen tube growth, accompanied by excessive deposition of plasma membrane and cell wall material at the tip, with RALF4 modulating cytoplasmic levels of ROS and Ca^2+^ in opposite ways at the tip. The authors state that RALF4 and RALF19 (RALF4/19) play a crucial role in maintaining pollen tube growth by interacting with the CrRLK1L family receptor-like kinases ANXUR1 and ANXUR2 (ANX1/2) and Buddha’s Paper Seal 1 and 2 (BUPS1/2) peptides [43,175], forming a complex with two pollen LORELEI-Like-Glycosylphosphatidylinositol (GPI)-anchored proteins (LLG2/3) [166,175] and the COBRA-like protein 11 (COBL11) [176]. These events activate a signaling pathway involving a receptor-like cytoplasmic kinase (RLCK) called MARIS (MRI) [79] and pollen-expressed NADPH oxidases (respiratory burst oxidase homologs H and J, RBOH H/J), regulating cytosolic ROS and calcium levels [177]. On the other hand, RALF4 also interacts with leucine-rich repeat extensins (LRX8/9/10/11), chimeric proteins involved in cell wall assembly during pollen tube growth [178]. Pollen from plants with knock-out and knock-down of the corresponding genes, including *ralf4-1* and *amiRRALF4/19*, bursts prematurely to varying degrees during growth. In contrast, overexpression of ANX1/2 suppresses pollen tube growth [167].

Data on the role of RALFs in pollination/fertilization processes in members of other plant families are rather scarce. Similar to their vegetative orthologs, the tomato (*Solanum lycopersicum* L.) pollen-specific gene *SlPRALF*, described in Covey et al. [64], appears to be a negative regulator of PT growth, as evidenced by the effect of synthetic SlPRALF peptide on tomato PT growth in vitro. The synthetic SlPRALF peptide did not affect pollen hydration or viability but suppressed the elongation of normal pollen tubes in an in vitro growth system. The fact that SlPRALF suppresses the growth of PT may at first seem surprising, since pollen itself produces this peptide. Pollen germination and pollen tube growth are known to be self-regulating processes. Furthermore, its action was reversible. Adding 100 nM SlPRALF to actively growing pollen tubes suppressed further elongation until the tubes reached a length of 40–60 mm, after which the pollen tubes became resistant to the peptide. This correlated with the time of the male germ unit’s exit from the pollen grain into the tube. Thus, the authors concluded that exogenous SlPRALF acts as a negative regulator of pollen tube elongation during a specific developmental period.

Kou et al. [179] identified PbrRALF2, a pear (*Pyrus bretschneideri*) pollen RALF peptide that inhibits pollen tube growth. The authors found that PbrRALF2 interacts with a malectin-like domain-containing RLK, PbrCrRLK1L13, which mediates PbrRALF2-induced ROS production. Excessive ROS accumulation inhibits pollen tube growth. Somoza et al. [174] showed that adding synthetic RALF4 in vitro increases H_2_O_2_ levels in the cytoplasm of the pollen tube tip, immediately halting its growth. This is consistent with data from Boisson-Dernier et al. [167] that *ANX1* overexpression is responsible for increased ROS production, leading to the arrest of pollen tube growth.

### 6.2. Interspecific Reproductive Barriers

The rejection of unwanted pollen on the stigma is crucial for preventing outcrossing but can be overcome under certain conditions, such as with mentor pollen support. Recent studies on *Brassica rapa* have shown that the stigmatic S-locus receptor kinase (SRK) receptor, known for regulating SI in *Brassicaceae*, promotes the rejection of both intraspecific and interspecific pollen via a FER-dependent ROS signal [180]. This discovery revealed that this SI system can serve as an interspecific hybridization barrier in crops containing SRK. It is logical to assume that the mechanisms of SI and Interspecific reproductive barriers (IRB) function should be similar and have a similar pollen recognition signaling system, but these points largely remain debatable [181].

Recently, Lan et al. [171] demonstrated that by manipulating the corresponding RALF peptide signals, as well as components of the CrRLK1L receptor complex and LRX cell wall proteins, IRB in *Brassicaceae* can be successfully overcome. This manipulation led to fertilization between distantly related species and the production of hybrid embryos. Furthermore, accumulating evidence suggests that leucine-rich repeat-extensin (LRX) proteins play a conserved role in regulating cell wall properties through interaction with RALF. It was demonstrated that LRX3, LRX4, and LRX5 can bind stigmatic RALFs, thereby creating a reproductive barrier. After self-compatible pollination, pollen RALFs can also bind to these LRX proteins, potentially disrupting LRX dimers and promoting the breakdown of the stigmatic barrier. In contrast, interspecific incompatible pollen could penetrate the stigmas of plants lacking LRX3, LRX4, and LRX5 proteins (Figure 6e) [171].

The “lock and key” system also operates here. In *Arabidopsis*, the receptor-like kinases FERONIA/CURVY1/ANJEA/HERCULES RECEPTOR KINASE1 and the cell wall proteins LRX3/4/5 interact with sRALF1/22/23/33 on the papilla cell surface, establishing a lock that blocks the penetration of unwanted pollen tubes. Compatible pRALF10/11/12/13/25/26/30 peptides act as a key, displacing sRALF and enabling pollen tube penetration. By treating *Arabidopsis* stigmas with synthetic pRALF, the barrier is unlocked, facilitating pollen tube penetration from distantly related *Brassicaceae* species and leading to the formation of interspecific/intergeneric hybrid embryos. Thus, the discovery of the “lock and key” system governing the hybridization breadth of interspecific/intergeneric crosses in *Brassicaceae* promises to facilitate wide hybridization in agricultural crops.

Lan et al. [171] also showed that pollen from distantly related species could only partially penetrate stigmas with a defective lock or after pRALF26 treatment, suggesting the presence of some additional mechanisms. A secondary penetration barrier may also serve to reject interspecific PTs in the *Brassicaceae* family. A total of seven *Arabidopsis* leucine-rich repeat malectin receptor kinases (LRR-MAL RKs), specifically RKF1, RKFL1-3, LysM RLK1-INTERACTING KINASE1, REMORIN-INTERACTING RECEPTOR1, and NEMATODE-INDUCED LRR-RLK2, were found to form a prezygotic IRB for *Capsella rubella* PT during penetration. It was reported that *Arabidops*is PTs germinated normally into the style, unlike *C. rubella* PTs, whose growth was impeded. This result shows that removing the stigmatic barrier in plants where this barrier is key can lead to fertilization and the development of hybrid embryos, thereby paving the way for successful interspecific/intergeneric hybridization.

### 6.3. Male and Female Gametophyte Interactions

Communication between PTs and synergid cells (SCs) during pollen tube reception is a complex process involving the interaction of both peptide and carbohydrate ligands with receptor complexes to generate gaseous and calcium downstream signals that ultimately control PT growth rate, direction, and rupture. Recent advances in understanding the role of RALF ligands produced in PTs represent an important step forward in identifying specific molecular players, but they raise new questions regarding the reasons for the high apparent redundancy of these proteins and the potential role of other RALFs, particularly as return signals from the ovule [182].

Although hundreds of PTs can germinate in the pistil’s transmitting tract, typically only one PT, in response to attractants, exits the septum in close proximity to each ovule to reach the embryo sac (Figure 6f). RALF4 and RALF19 from the PT signal actin autocrinally to maintain PT integrity by binding to the ANX1/2–BUPS1/2 complex at the PT tip. RALF34 is expressed throughout the ovule and in vitro readily displaces RALF4 and RALF19 from binding to the receptor complex, triggering PT rupture [25]. Despite very low concentrations of RALF34 triggering PT rupture in vitro, *ralf34* mutant plants have no fertility defect, indicating that this peptide is not the sole signal capable of inducing PT rupture.

Zhong et al. [46] note that RALF4/19 are necessary not only for maintaining proper PT growth, at least in vitro, but they are also involved in PT reception by SCs. Synthetic RALF4 binds to the FER–LRE–NTA protein complex of the filiform apparatus and triggers Ca^2+^ release in the SC cytoplasm, similar to how arriving PTs act. Other pollen-expressed RALFs (RALF6, 7, 16, 36, 37) have also been reported to interact in vitro with FER, ANJEA (ANJ), and HERCULES RECEPTOR KINASE1 (HERK1) [45]. One of them, RALF37, like RALF4 and RALF19, also triggers Ca^2+^ release in the SC and activates the FER–LRE–NTA protein complex [21]. Yu et al. [183] showed that ovule-expressed RALF34 induces PT rupture in vitro and binds in vitro to ANX1/2 and BUPS1/2, displacing RALF4/19. Given that RALF34 binds to FER, at least in vitro, but cannot increase [Ca^2+^]cyt activity or that of FER-LRE-NTA, its function remains unclear.

In *Arabidopsis thaliana*, the prevention of polyspermy is ensured by a mechanism that prevents polytubey (the entry of multiple PTs into one ovule). Zhong et al. [46] showed that the receptor-like kinases FERONIA, ANJEA, and HERCULES RECEPTOR KINASE 1, located in the septum, interact with PT-specific peptide ligands RALF6, 7, 16, 36, and 37 to create this polytubey block (Figure 6f). The same combination of RALF peptides and receptor complexes controls PT reception and its rupture inside the target ovule. PT rupture releases the polytubey block in the septum region, allowing secondary PTs to form if fertilization fails. Thus, the organized stages of the fertilization process in *Arabidopsis* are coordinated by the same signaling components, ensuring and optimizing reproductive success.

Zhong et al. [46] used CRISPR-Cas9 to generate *ralf36 ralf37* double mutants, *ralf6 ralf7 ralf16* triple mutants, and *ralf6 ralf7 ralf16 ralf36 ralf37* quintuple mutants. All exhibited normal vegetative growth. When pistils of wild-type plants were pollinated with mutant pollen, all three *ralf* mutants displayed a polytubey phenotype, resembling that observed in *fer-4*, *anj herk1*, or *fer anj herk1* mutant pistils pollinated with wild-type pollen. The higher ratio of polytubey caused by the quintuple *ralf* mutation compared to double or triple *ralf* mutations suggests that the five RALF genes act together to form the polytubey block. Therefore, the authors proposed that all five RALF peptides are signaling molecules produced by the PT, which are likely perceived by the FER-ANJ-HERK1 receptor complex in the septum, forming the polytubey block, and suggested the following model (Figure 6f). PT attractants secreted by the SC stimulate the nearest PT to exit the transmitting tract. RALFs secreted by this PT activate FER, ANJ, and HERK1 signaling pathways in the epidermal cells of the septum, establishing a polytubey block that prevents the exit of additional PTs. This male-female formed polytubey block remains activated during the PT’s growth into the ovule due to the continuous production of RALF peptides by the first-arrived PT. After successful recognition by the same receptor complex (FER-ANJ-HERK1) in the SCs, the PT ruptures, releasing the two sperm cells. Upon completion of fertilization, the polytubey block is removed as RALFs quickly disappear from the ruptured PT.

The results of Gao et al. [45] complement the data from Zhong et al. [46], as these two studies tested mutually exclusive lists of RALF peptides. Gao et al. [45] investigated the interaction of eight pollen tube-expressed RALFs and the ovule-expressed RALF34 with FER and concluded that among them, only RALF4, RALF19, and RALF34 bind to FER, and that the presence of any of these three RALFs enhances the interaction of FER with the LORELEI (LRE) coreceptor [45]. Collectively, these two recent studies show that several RALF peptides can act as signals from the pollen tube to the synergid cells during pollen tube reception. The reason for such functional redundancy remains unclear, but it may be necessary to ensure the robustness of the response.

## 7. Primary Root Regulation

One of the initial functions discovered for RALFs peptides is the inhibition of primary root elongation [184]. The inhibitory effect of RALF1 in *Arabidopsis* was demonstrated experimentally, where overexpression of AtRALF1 resulted in bushy, semi-dwarf plants with small leaves, short roots, and a reduced number of lateral roots and small root cells [66,185]. On the other hand, in *Arabidopsis* with *AtRALF1* gene knockdown, the opposite effect was observed: plants exhibited root and hypocotyl elongation, and the number of lateral roots and large root cells increased [185]. The hypotrophic effect in tomato and Arabidopsis was demonstrated using a synthetic RALF1 peptide [16]. However, it should be noted that most RALF family peptides have a negative effect on root development. One of the known functions of RALF is to prevent cell wall acidification by blocking membrane-bound proton pumps, which leads to rapid alkalization of the environment [186]. AtRALF1 was shown to increase the Ca^2+^ concentration in the cytoplasm of Arabidopsis thaliana root cells, suggesting that RALF peptides likely mediate a Ca^2+^-dependent signaling pathway [187]. Thus, RALF peptides play a key role in physiological cellular processes and are likely involved in the regulation of cell expansion [16,64,66,67,76].

**Figure 7:**
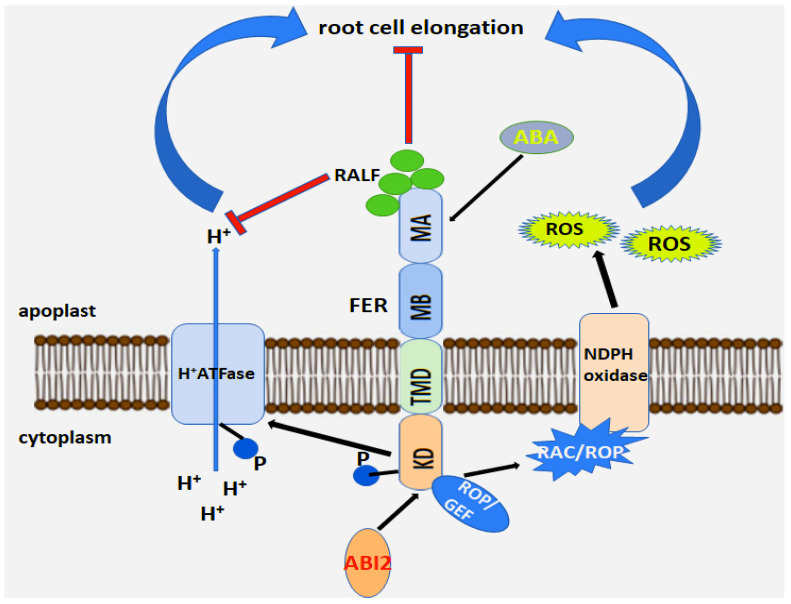
Schematic representation of the role of RALF-FER in the ABA signaling pathway. RALF and ABA activate FER phosphorylation. Phosphorylated FER regulates ROS production via the GEF1/4/10–ROP11/ARAC10 pathway. Conversely, phosphorylated FER leads to activation of ABI2, which dephosphorylates FER, suppressing the FER–GEF–ROP pathway.

The mechanisms by which AtRALF1-FER signaling regulates root growth remain unclear. Several mechanisms for the suppression of root growth by the RALF1 peptide have been proposed. One mechanism has been proposed based on the fact that AtRALF1 controls root growth suppression through the formation of a complex with the FER receptor. Chen et al. [39] identified several factors that can activate FER. They observed that the RALF peptide and the hormone ABA activate FER, resulting in phosphorylation of the FER receptor. Phosphorylation of FER leads to root growth suppression. Conversely, protein phosphatase (ABI2) acts as a negative regulator of ABA signaling, dephosphorylating FER, thereby providing a critical feedback mechanism for ABA-induced FER activation, resulting in the observed activation of primary root growth. In addition, another pathway to suppress root growth via ABA has been proposed. Based on this mechanism, the hormone, by binding to its receptors, activates SnRK2, which can directly inhibit AHA2-mediated acidification, resulting in inhibition of root growth [188,189,190]. In contrast, the peptide hormone RALF binds to the FER receptor and activates it to directly inhibit AHA2-mediated root growth [6]. Furthermore, in a “cross-talk” mode, RALF activates ABI2 via the GEF–ROP pathway with its receptor FER. GEF–ROP activation is accompanied by feedback inhibition of the ABA response [191]. However, using the *fer-4* mutant, which disrupts the RALF–FER pathway, as well as a knockout of the *RALF* gene, an enhanced response to ABA was observed. This is consistent with results showing that RALF addition reduces the response to ABA in the root growth assay (Figure 7). Conversely, ABA addition enhances the response to RALF, consistent with the model in which ABA inhibits AHA2, which suppresses FER activity. Since H^+^-ATPases (AHAs) are also regulated by other factors (e.g., auxin), other mechanisms may exist that regulate root growth through AHA-mediated acidification of the extracellular space.

Another hormone, brassinolide (BL), has also been found to be involved in the regulation of primary root growth. Brassinosteroids (BRs), of which BL is a member, are important regulators of growth and development and play a key role in cell division, hypocotyl elongation, and root growth [192]. BR-mediated cell expansion involves proteins that modify the cell membrane and reorient cortical microtubules. BL activity is mediated by the leucine-rich repeat receptor kinase BRI1. Binding of BR to BRI1 (BRASSINOSTEROID INSENSITIVE1) triggers autophosphorylation of the kinase domain and subsequent recruitment of the co-receptor BRI1-ASSOCIATED KINASE1 (BAK1), resulting in the activation of BR-responsive genes [192,193]. AtRALF1 also binds to BAK1, and BAK1 plays a key role in the suppression of root growth and cell division. Antagonism between AtRALF and BR was demonstrated by identifying genes involved in cell wall remodeling that were induced by AtRALF [185,194]. It was shown that the antagonistic relationship between AtRALF and BR can be regulated by turning on and off both signaling pathways through BAK1 [40].

## 8. RALF-FER Regulate Root Hairs

Root hairs (RH) play an important role in water and nutrient uptake from the soil [195]. A number of genetic factors are involved in the regulation of root hair differentiation and growth, such as ROOT HAIR DEFECTIVE6 (RHD6), NADPH oxidase that produces ROS, which stimulate Ca^2+^ ion uptake at the tips of growing root hairs [196,197] and EXPANSIN7 (EXP7) proteins, which are involved in cell elongation [198]. RHD6 is a basic helix-loop-helix (bHLH) transcription factor that determines the position of root hair exit, regulates hair cell elongation by controlling the expression of hundreds of genes [199,200]. RHD-6-LIKE 4 (RSL4) encodes a bHLH transcription factor [200]. RH growth duration and size must be tightly regulated. Several mechanisms for regulating the synthesis of proteins encoded by these RH-associated genes have been proposed.

**Figure 8:**
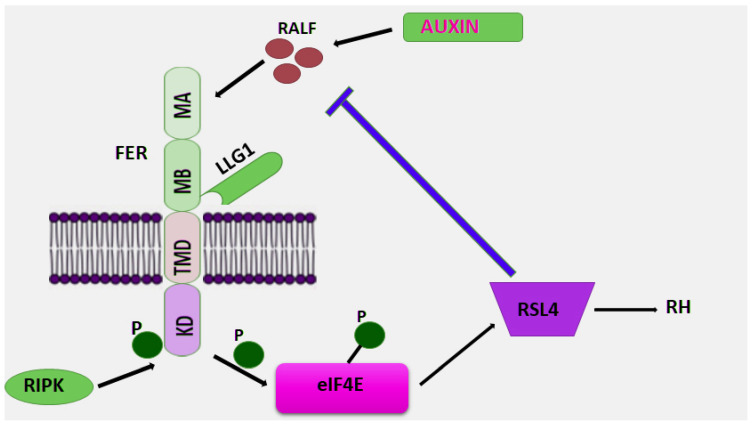
Schematic representation of the role of FER in auxin-induced root hair growth. Auxin activates the binding of FER to the RALF peptide, leading to phosphorylation by the KD kinase RIPK. This is followed by phosphorylation of eIF4E (eukaryotic translation initiation factor 4E) and activation of the *RSL4* gene (ROOT HAIR DEFECTIVE 6-LIKE 4), which activates root hair growth. At high concentrations of the RALF peptide, RSL4 inhibits its biosynthesis. Thus, a negative feedback loop, RALF-FER-RSL4, is formed, regulating root hair development.

An autocrine mechanism responsible for the regulation of RH growth has been proposed, in which the extracellular peptide RALF1 and the receptor FER act as a central hub between the cell surface and downstream signaling pathways [201]. RALF1 is secreted into the apoplast, binds to the FER kinase localized on the surface membrane, and influences the size of *Arabidopsis* rhesus cells (Figure 8) [96,202]. Formation of the RALF1-FER complex triggers the recruitment of RPM1-induced protein kinase (RIPK). Mass spectrometry showed that inducible RIPK binds to FER upon treatment with RALF1 [96]. The *ripk* mutant exhibited a short root hair phenotype and was insensitive to RALF1 treatment, similar to *fer-4*. Meanwhile, overexpression of RIPK rescued the root hair defects in the *fer-4* mutant, suggesting that FER and RIPK may cooperatively control root growth (Figure 8) [96]. The interaction between RIPK and FER was also supported by biochemical studies. This suggests that the ligand RALF1 binds to FER, further recruiting RIPK to the kinase complex and triggering a phosphorylation cascade that leads to environmental alkalinization and inhibition of root growth [6,202]. RALF1 treatment has been shown to induce interdependent phosphorylation of both FER and RIPK [202].

Protein phosphorylation results in activation of the early translation initiation factor (eIF4E1) followed by its phosphorylation [202]. Phosphorylation enhances the ability of eIF4E1 to bind to mRNA and increases the rate of synthesis of specific RH proteins, such as NOXC, PRX73, ROP2, including RSL4 [202]. RALF1, FER, and eIF4E1 are localized in the polar regions of ribosomes, providing spatial control of local protein synthesis and determining the final cell size. As a result of this mechanism, RSL4 levels increase, but on the other hand, high RSL4 levels lead to inhibition of RALF1 mRNA expression by directly binding to the RALF1 promoter, which negatively affects the RALF1–FER signaling pathway [202]. This slows down RH growth and determines the final RH cell size. Thus, a negative RH growth regulation loop is formed. This suggests that a precise regulatory mechanism controls cell size and polar growth through transmembrane receptors associated with extracellular signals such as RALF peptides.

Auxin is a key regulator of RH growth. The hormone stimulates the binding of several auxin response factors (ARFs) to AuxRE sites on the RSL4 promoter, thereby increasing *RSL4* expression. Several mechanisms have been described to explain the function of FER in auxin-regulated root hair growth. One is that FER regulates NADPH oxidase-dependent RHO GTPase (RAC/ROP)-mediated ROS accumulation through interaction with the RHO GTPase guanine nucleotide exchange factor (ROPGEF) (Figure 7) [93]. Plant RHO GTPases (RAC/ROP) transduce various extracellular signals, from hormonal to stress, and regulate various cellular processes important for polarized cell growth, differentiation, development, proliferation, and their response to the environment. They switch between an inactive GDP-bound state and an activated GTP-bound state, and their activation is predominantly mediated by a family of guanine nucleotide exchange factors (GEFs) called ROPGEFs. Following signal activation, activated RAC/ROP bound to GTP (guanine triphosphate) interacts with NADPH oxidase, resulting in ROS production and root hair growth [93]. FER interacts with the GTPase ROP2 and acts upstream of RAC/ROP signaling to regulate ROS production and root hair growth. However, the exact mechanism of this process is unclear. Moreover, the role of FER in this mechanism remains unclear. A key question that remains unclear is whether phosphorylation of the GTPase enzyme ROP2 occurs upon FER binding and whether complex formation with FER affects the stability or function of the ROP2 GTPase. However, it has been noted that a decrease in functional FER disrupts the balance between inactive and activated forms of RAC/ROP. Disruption of the balance between different forms of RAC/ROP signaling results in a significant reduction in ROP GTPase-mediated ROS production in root hairs [203,204]. Furthermore, other FER-related factors may also be involved in this process. It has been reported that the glycosylphosphatidylinositol-binding protein (GPI-AP), LRE-like GPI-AP1 (LLG1), forms a signaling complex with FER–RHO GTPase and is its key component [100]. In particular, LLG1, by forming a complex with FER, acts as a “chaperone”, transporting FER from the endoplasmic reticulum (ER) to the cytoplasmic membrane, where FER functions [100]. FER has been shown to remain localized to the ER following LLG1 loss and the development of a root hair growth phenotype similar to the *fer* mutant, suggesting a key role for the LLG1-FER-ROPGEF-RAC/ROP signaling complex [93,100].

Using *Arabidopsis* ROPGEF1 as a “bait,” members of the receptor-like kinase (RLK) family were identified as potential upstream regulators of RAC/ROP signaling. ROS generated by NADPH oxidase are emerging as important regulators of growth and development and play a key role in RAC/ROP-driven root hair development, a process of polarized cell growth. T-DNA insertion mutants in these RLKs were tested for root hair defects, and mutations in one of them, *At3g51550*, encoding the receptor-like kinase FER, were found to cause severe root hair defects. The *fer* phenotypes were shown to correlate with reduced levels of active RAC/ROP and accumulation of auxin-regulated, NADPH oxidase-dependent ROS in roots and root hairs. Collectively, these observations strongly suggest that FER is a regulator of the RAC/ROP signaling pathway that controls ROS-induced root hair development. Furthermore, *FER* expression was downregulated by ROP2 GTPase, consistent with regulation of guanine nucleotides, suggesting the existence of a dynamic signaling complex involving FER, ROPGEF, and RAC/ROP.

A second possible mechanism for regulating root hair growth is that auxin indirectly modulates apoplast acidification, stimulating root cell growth (Figure 8). By binding to its ligand RALF, FER induces phosphorylation of plasma membrane-localized H^+^-ATPase, resulting in inhibition of proton transport into the apoplast [6]. Numerous studies have shown that apoplast pH plays a key role in regulating local cell growth [205,206]. Barbez et al. [91] also reported that decreased auxin levels, auxin perception, or signaling strongly affect apoplast acidification and cell growth [91]. However, increased levels of exogenous and endogenous cellular auxin result in transient alkalization of the apoplast, which in turn leads to FER-dependent suppression of root cell growth. The interaction of FER with its ligand RALF1 triggers phosphorylation of the plasma membrane-localized proton pump (H^+^-ATPase) [6]. Following phosphorylation, the proton-transfer activity of H^+^-ATPase is inhibited, leading to subsequent alkalinization of the apoplast [6]. It has also been shown that functional loss of FER leads to the abolition of auxin-induced alkalinization of the apoplast, which, in turn, leads to a decrease in root cell growth [91]. These results confirm the close association of FER with auxin-mediated cell elongation through the regulation of apoplast pH. ROS is an important second messenger in the ABA signaling pathway [92]. The FER–ROPGEF–ROP–NADPH oxidase pathway, mediated by ROS production, is involved in auxin-mediated root hairs, is also involved in the ABA signaling pathway. ROS play a key role in ABA-mediated stomatal closure [92]. Disruption of FER function results in the stomatal gap becoming smaller than that of the wild type upon ABA treatment, which may be caused by the high ROS content in the mutant *fer* gene [43]. Thus, FER regulates ROS production via the GEF–ROP pathway, participating in plant cell elongation mediated by both ABA and auxin.

Recently, it has been shown that ectopic expression of *RSL4* driven by the GLABRA2 (GL2) promoter induces RH growth in *Arabidopsis* atrichoblasts, which do not normally produce RHs [207]. This suggests that *RSL4* expression alone can induce ectopic RH response [207]. Furthermore, *RSL4* controls the expression of hundreds of genes. Collectively, these properties make *RSL4* a master regulator of Rh cell growth and, consequently, final cell size. Recently, more reports have emerged on the genetic components regulating RH growth, which regulate the expression of *RHD6*, *RSL4*, and *RSL2* to a certain extent and thus inhibit RH growth [208]. Similarly, *GT-2-LIKE 1* (*GTL1*) and its homolog *DF1* bind to the RSL4 promoter and suppress RH growth [209]. Multiple regulatory levels may coordinately control *RSL4* transcriptional activation during RH growth.

As a result of the transcriptional auxin response, *RSL4* expression increases several-fold, linking auxin stimulation to *RSL4* expression at the molecular level [201]. During RH growth, ROS homeostasis is required to balance cell wall rigidity through cross-linking and loosening of the extensin network, as well as ROS-mediated stimulation of plasma membrane Ca^2+^ channels [210]. RALF1 may also interact with other receptors. RALF1 has been shown to stimulate phosphorylation of plasma membrane H^+^-adenosine triphosphatase 2 (AHA2), which inhibits proton transport, causing a decrease in cell elongation [200]. Interestingly, PSY1 and RALF exert opposite effects on plant growth by differentially regulating membrane-localized AHA2 [6,211]. The RALF34 peptide is perceived by another CrRLK1L member, THESEUS1 (TE1), to fine-tune pericycle cell division patterns during lateral root initiation [50,212]. *ralf34* and *the1* mutants exhibited comparable lateral root phenotypes, and the1 mutant was insensitive to RALF34-induced root growth inhibition and surface alkalinization [48]. FER also plays a key role in maintaining actin cytoskeletal integrity by mediating the polar localization of PIN-forming protein 2 (PIN2), which promotes lateral root development and gravitropic responses [213].

## 9. Conclusions

More than 20 years have passed since the discovery of RALF1. During this time, 37 members of the RALF family have been identified, but the functions and tissue localizations of many family members remain unknown. Of all the known family members, RALF1 lacks tissue specificity and is localized in all plant tissues. It is possible that peptides localized in the same tissue and sharing homologous amino acid sequences complement its functions when needed, for example, in the presence of a strong and prolonged external signal. Furthermore, peptides from other clades can perform the functions of RALF1 during its inactivation. It is conceivable that different signals from external stress factors trigger the activation of different RALF family members.

Studies show that RALF effects are context-dependent and vary depending on plant species, developmental stage, and stress type. This complexity requires further research to identify the specific RALFs and their corresponding FERONIA receptors involved in specific stress responses. Identifying these peptide-receptor pairs will enable the development of more precise and effective strategies for manipulating plant stress responses. It remains unclear what the primary stimulus is for their binding: activation of RALF peptides by hormones, binding of peptides to stress ligands, or activation of FER by coreceptors such as LLG1. It is unclear how many RALF molecules interact with FER, whether there are differences in the binding sites of different RALF family members (not only those with the YISY motif), how the kinase domain is activated upon RALF binding, and if there is a relationship between FER phosphorylation levels and binding to different RALF family members. FER phosphorylation also depends on the ligand bound to the RALF peptide. This is one of the important factors that control signaling and attract other coreceptors. Interactions with coreceptors are fundamental factors controlling the cell wall, the penetration of signaling molecules, and the formation of a signaling cascade. Several studies indicate direct monitoring of cell wall status by FER, but it remains unknown whether FER directly senses the cell wall ligand or detects changes in cell wall mechanics. Equally interesting is the question of intracellular signal transduction during the internalization of FER-specific proteins induced by RALF. The cascade pathways involved in signal transduction that regulate gene expression in response to various external signals remain to be explored. Understanding the regulation of the complex signaling mechanisms of the RALF-FER complex will require experimental approaches with high spatial and temporal resolution. Future research should aim to decipher the complex signaling pathways involving RALF-FER, particularly the mechanisms of interaction with coreceptors, the influence of cell wall modifications, and the role of intracellular signaling. Using approaches with high spatial and temporal resolution will provide valuable insights into the dynamics of these complexes and the regulation of the corresponding genes. Understanding the precise molecular mechanisms underlying the RALF-FERONIA interaction is crucial for unlocking the potential of RALF as a tool for enhancing plant stress tolerance and optimizing growth. By modulating specific signaling pathways activated by RALF, it is possible to specifically influence plant physiological processes, improving their adaptation to various environmental conditions.

Importantly, the role of RALF is not limited to signaling through FERONIA. Recent discoveries regarding the structural functions of RALF in the cell wall open new perspectives for understanding the influence of these peptides on plant growth and development. The interaction of RALF with demethylated pectin via extensins can influence the mechanical properties of the cell wall, determining the shape of cells and tissues, as well as their ability to withstand mechanical stress.

Further research is also needed to elucidate the precise nature of the ligand-receptor specificity between RALF and FER in the context of plant immunity. It is important to determine which specific RALF isoforms most effectively activate FER in response to pathogen attack and how this activation translates into specific defense responses. Understanding these subtleties will enable the development of strategies for targeted manipulation of RALF-FER signaling pathways to enhance plant disease resistance.

Along with elucidating ligand-receptor specificity, it is necessary to further understand the intracellular signaling events triggered by the RALF-FER complex. Identifying and characterizing the downstream components that interact with FER after RALF binding will provide a comprehensive understanding of the molecular mechanism underlying RALF-FER-mediated immunity. Plants likely utilize a fine-tuning system to prioritize the various processes involving FER depending on the current situation. A deeper understanding of these interactions will enable more effective use of RALF-FER to improve plant health and yield.

Research on RALFs has primarily focused on the model plant Arabidopsis. However, studying their role in agricultural crops is of great interest. Given their involvement in global signaling from external stressors, the alleviation of self-incompatibility, and the facilitation of distant hybridization, RALF peptides may become promising adaptogens and growth regulators for enhancing the resilience and yield of agricultural plants. Detailed study of RALFs in agronomically important crops could reveal species-specific variations in their functions and interactions, opening new avenues for improving agricultural traits.

In recent years, significant advances in gene editing technologies such as CRISPR-Cas9 have provided tools for the precise manipulation of genes encoding RALF peptides. This allows for the creation of edited versions of RALFs with redundant or altered functions tailored to specific agricultural applications. Furthermore, advances in biotechnology offer prospects for the industrial production of active RALF peptides, which could facilitate the development of new agrochemicals to enhance plant stress tolerance and improve crop yields.

Overall, RALF family peptides represent a multifaceted regulator, a promising area of research with the potential for significant advances in understanding plant growth processes, stress tolerance, and reproductive biology. The use of modern tools and technologies, such as gene editing and high-throughput screening, could pave the way for the development of innovative strategies to improve crop productivity and resilience.

## Figures and Tables

**Table 1:** Members of RALF peptide family.

Name	Localization	Regulation	References
RALF1	Root	Root growth inhibition	[6,39,40]
		Extracellular alkalinizing activity	
		Inhibit elf18-induced ROS	[21]
		Affect the flowering time	[41]
		Negative regulation of salt tolerance	[42]
RALF2	Pollen, seed, leaf hypocotyl	Unknown	
RALF3	Pollen, seed	Unknown	
RALF4	Pollen tube Synergids	Maintaining the integrity of pollen tubes	[25,43,44]
		Triggered Ca^2+^ oscillation	[45]
		Inhibit elf18-induced ROS	[21]
RALF5	Pollen, seed	Unknown	
RALF6	Pollen and pollen tubes Root	Controls the polytubey block	[46]
		Root growth inhibition	[21]
		Increase elf18-induced ROS	[21]
RALF7	Pollen and pollen tubes Root	Controls the polytubey block	[46]
		Root growth inhibition	[21]
		Extracellular alkalinizing activity	[21]
		Increase elf18-induced ROS	[21]
RALF8	Root	Root growth inhibition	[21]
		Increase elf18-induced ROS	[21]
		Negative regulation of drought tolerance	[47]
RALF9	Root	Root growth inhibition	[21]
		Increase elf18-induced ROS	[21]
RALF10		Increase elf18-induced ROS	[21]
RALF11	Unknown	Unknown	
RALF12	Unknown	Unknown	
RALF13		Increase elf18-induced ROS	[21]
RALF14	Unknown	Unknown	
RALF15	Root	Root growth inhibition	[21]
RALF16	Root	Root growth inhibition	[21]
		Extracellular alkalinizing activity	
		Increase elf18-induced ROS	[21]
RALF17	Pollen and pollen tubes Root	Controls the polytubey block	[46]
		Root growth inhibition	[21]
		Increase elf18-induced ROS	[48]
RALF18	Unknown	Unknown	
RALF19	Root pollen tubes Synergids	Root growth inhibition	[21,49]
		Extracellular alkalinizing activity	
		Maintaining the integrity of pollen tube	[25,43,44]
		Triggered Ca^2+^ oscillation	[45]
RALF20	Root	Root growth inhibition	[21]
		Increase elf18-induced ROS	[21]
RALF21	Root	Root growth inhibition	[21]
RALF22	Root	Root growth inhibition	[49,50]
		Extracellular alkalinizing activity	
		Negative regulation of salt tolerance	[51]
		Inhibit elf18-induced ROS	[21]
RALF23	Root	Root growth inhibition Extracellular alkalinizing activity Inhibit elf18-induced ROS Negative regulation plant immunity Negative regulation of salt tolerance Promotion of *Pseudomonas* Colonization Pollen-Stigma Involvement in affinity pollination mechanisms	[21,50] [48,52] [51] [53] [54]
RALF24	Root	Root growth inhibition	[21]
		Increase elf18-induced ROS	[21]
		Extracellular alkalinizing activity	[49]
RALF25	Unknown	Unknown	
RALF26	Unknown	Unknown	
RALF27	Unknown	Unknown	
RALF28	Unknown	Unknown	
RALF29		Increase elf18-induced ROS	[21]
RALF30		Increase elf18-induced ROS	[21]
RALF31	Root	Root growth inhibition	[49]
		Extracellular alkalinizing activity	
		Increase elf18-induced ROS	[21]
RALF32	Root	Root growth inhibition	[21]
		Increase elf18-induced ROS	[48]
RALF33	Root Pollen-Stigma	Root growth inhibition Extracellular alkalinizing activity Inhibit elf18-induced ROS Promotion of *Pseudomonas* colonization Involvement in affinity pollination mechanisms	[50,55] [48] [53] [54]
RALF34	Root Ovules	Primary lateral root development	[50]
		Inhibit elf18-induced ROS	[48]
		Root growth inhibition	[21,49]
		Extracellular alkalinizing activity	
		Induction of pollen tube rupture and sperm release	[25]
RALF35	Root	Root growth inhibition	[21]
		Increase elf18-induced ROS	[21]
RALF36	Root Pollen and pollen tubes	Root growth inhibition	[21]
		Extracellular alkalinizing activity	
		Increase elf18-induced ROS	
		Controls the polytubey block	[46]
RALF37	Pollen and pollen tubes	Controls the polytubey block	[46]

The Arabidopsis RALF34 peptide has been suggested to be involved in the regulatory network controlling lateral root formation [31].

## Data Availability

No new data were created or analyzed in this study. Data sharing is not applicable to this article.

## Abbreviations

ABA	abscisic acid
ABI2	ABA insensitive 2
AHA2	H^+^–ADENOSINE TRIPHOSPHATASE 2
BR	brassinosteroid
BZR1	BRASSINAZOLE RESISTANT 1
CDPK	Calcium-dependent protein kinases
CrRLK	Catharanthus roseus receptor-like kinase
eIF4E	eukaryotic translation initiation factor 4E
elf18	elongation factor (first 18 amino acids)
FER	FERONIA receptor
GEF	plant-specific signaling molecules
LLG1	LORELEI-LIKE GLYCOSYLPHOSPHATIDYLINOSITOL-ANCHORED PROTEIN 1
LRE	LORELEI member of the LLG family
LRK**s**	Leucine repeat receptor kinases
LRX	leucine-rich repeat extensins
NADPH	nicotinamide adenine dinucleotide phosphate
PCP-B	POLLEN COAT PROTEIN B-class
PG	pollen grain
PT	pollen tube
RAC/ROP GTPases	plant-specific signaling molecules
RALF	Rapid alkalinization factor
RIPK	RESISTANCE INDUCED PROTEIN KINASE
RBOH	Respiratory burst oxidase homolog
ROPGEF	the guanine nucleotide exchange factors that activate RAC/ROPs
ROS	reactive oxygen species
RSL4	ROOT HAIR DEFECTIVE 6-LIKE 4
SCR	S-locus receptor kinase
